# Strengthening and Sustaining Inter-Institutional Research Collaborations and Partnerships

**DOI:** 10.3390/ijerph18052727

**Published:** 2021-03-08

**Authors:** Jerris R. Hedges, Karam F. A. Soliman, William M. Southerland, Gene D’Amour, Emma Fernández-Repollet, Shafiq A. Khan, Deepak Kumar, Cecilia M. Shikuma, Brian M. Rivers, Clayton C. Yates, Richard Yanagihara, Winston E. Thompson, Vincent Craig Bond, Sandra Harris-Hooker, Shelia A. McClure, Elizabeth O. Ofili

**Affiliations:** 1 Department of Medicine and Surgery, John A. Burns School of Medicine, University of Hawaii at Manoa, Honolulu, HI 96813, USA; 2 Department of Basic Pharmaceutical Sciences, College of Pharmacy and Pharmaceutical Sciences, Florida Agricultural and Mechanical University, Tallahassee, FL 32307, USA; karam.soliman@famu.edu; 3 Department of Biochemistry & Molecular Biology, College of Medicine, Howard University, Washington, DC 20059, USA; wsoutherland@howard.edu; 4 Office of the President, Xavier University of Louisiana, New Orleans, LA 70125, USA; gdamour@xula.edu; 5 Department of Pharmacology & Toxicology, University of Puerto Rico Medical Sciences Campus, San Juan, PR 00936, USA; e.fernandez@upr.edu; 6 Department of Biological Sciences, Clark Atlanta University, Atlanta, GA 30314, USA; skhan@cau.edu; 7 Julius L. Chambers Biomedical/Biotechnology Research Institute, North Carolina Central University, Durham, NC 27707, USA; dkumar@nccu.edu; 8 Department of Medicine and Hawaii Center for AIDS, John A. Burns School of Medicine, University of Hawaii at Manoa, Honolulu, HI 96813, USA; shikuma@hawaii.edu; 9 Cancer Health Equity Institute, Morehouse School of Medicine, Atlanta, GA 30310, USA; brivers@msm.edu; 10 Department of Biology and Center for Cancer Research, Tuskegee University, Tuskegee, AL 36088, USA; cyates@tuskegee.edu; 11 Department of Pediatrics, John A. Burns School of Medicine, University of Hawaii at Manoa, Honolulu, HI 96813, USA; ryanagih@hawaii.edu; 12 Department of Physiology and Obstetrics & Gynecology, Morehouse School of Medicine, Atlanta, GA 30310, USA; wthompson@msm.edu; 13 Department of Microbiology, Biochemistry & Immunology, Morehouse School of Medicine, Atlanta, GA 30310, USA; vbond@msm.edu; 14 Department of Pathology & Anatomy, Morehouse School of Medicine, Atlanta, GA 30310, USA; sharris-hooker@msm.edu; 15 Office of Research Development, Morehouse School of Medicine, Atlanta, GA 30310, USA; smcclure@msm.edu; 16 Department of Medicine, Morehouse School of Medicine, Atlanta, GA 30310, USA; eofili@msm.edu

**Keywords:** RCMI, inter-institutional, collaborations, partnerships

## Abstract

Inter-institutional collaborations and partnerships play fundamental roles in developing and diversifying the basic biomedical, behavioral, and clinical research enterprise at resource-limited, minority-serving institutions. In conjunction with the Research Centers in Minority Institutions (RCMI) Program National Conference in Bethesda, Maryland, in December 2019, a special workshop was convened to summarize current practices and to explore future strategies to strengthen and sustain inter-institutional collaborations and partnerships with research-intensive majority-serving institutions. Representative examples of current inter-institutional collaborations at RCMI grantee institutions are presented. Practical approaches used to leverage institutional resources through collaborations and partnerships within regional and national network programs are summarized. Challenges and opportunities related to such collaborations are provided.

## 1. Introduction

The Research Centers in Minority Institutions (RCMI) program, currently administered by the National Institute on Minority Health and Health Disparities (NIMHD), has historically provided funding to build research infrastructure at resource-limited academic institutions serving minority communities [1]. The RCMI grantee institutions have demonstrated significant success in fostering basic biomedical and translational research and investigator development, through such National Institutes of Health (NIH) infrastructure support [2]. The RCMI program has recently transitioned from a G12 funding mechanism to U54 RCMI Specialized Centers (RCMI Centers), focusing on basic, clinical and behavioral research impacting the science of minority health and health disparities. These RCMI Centers are expected to build intra- and inter-institutional research collaborations and strengthen partnerships with community-based organizations to leverage this NIH investment. Specifically, scientists at these RCMI Centers are to develop research careers emphasizing successful collaborations. Scientific leaders at the RCMI Centers are expected to develop “standards and other mechanisms to maximize interoperability between internal systems and systems in external organizations…. likely to enhance collaborations with other research consortia and networks” [3].

The principal role of collaborations at RCMI Centers is to leverage existing research resources, strengthen investigator development and productivity, expand research opportunities, and facilitate inter-disciplinary and inter-institutional problem-solving. Successful team science demands effective collaboration to gain needed resources and expertise. Indeed, research impact upon a given discipline appears increasingly associated with domestic and international scientific collaboration [4,5]. The ability to establish productive research collaborations is one of the measures proposed to evaluate RCMI Center success [6].

A recent comprehensive review of multi-institutional collaborations focused on measuring the quality of the team science and outcomes of inter-institutional research collaborations [7]. Unfortunately, the mechanics of establishing inter-institutional collaborations were not addressed. A recent report using structured interviews with four selected Clinical and Translational Science Awards (CTSA) institutions identified some challenges and best practices experienced by these institutions regarding fiscal and administrative processes impacting collaborations with community partners, but not specifically with resource-limited RCMI Centers [8].

Given the importance of establishing and sustaining inter-institutional research collaborations and partnerships, we report investigators’ experience at established RCMI Centers regarding inter-institutional collaborations with majority-serving institutions. This collective experience can provide guidance of value to research leaders at resource-limited institutions seeking to build collaborations and partnerships with more resource-intense, majority-serving institutions.

## 2. Methods

Information regarding the nature of inter-institutional collaborations and partnerships at six selected RCMI Centers was obtained (5.0 Appendix A). The RCMI Center investigators were asked to summarize areas of major inter-institutional collaborations; how inter-institutional resources were used to leverage national research network programs; and the most important outcomes of these collaborations (e.g., mentorship, conferences, publications, presentations, and/or other grant funding). These summaries were presented at a special workshop during the 2019 RCMI Program National Conference on Collaborative Solutions to Improve Minority Health and Reduce Health Disparities, held in Bethesda, Maryland, on 15–17 December 2019.

Comments from the 2019 RCMI Program National Conference panel discussion were incorporated to summarize how the inter-institutional collaborations emphasized a health disparities focus within the national research network programs; how the national programs helped promote junior investigators; and how institutions addressed programmatic, operational, fiscal, and reporting barriers in the national programs which challenged the RCMI Centers’ ability to create synergy in scientific research and investigator development.

## 3. Panel Discussion on Inter-Institutional Collaborative Research

The RCMI Centers often lack a critical mass of extramurally funded subject-matter experts in key research areas and may have deficiencies in specific technical core instrumentation and services. These deficiencies may impact the competitiveness of institutional-based grants and the development of junior investigators at such resource-limited institutions, despite strong institutional connection to minority communities. This interactive panel discussion addressed the approaches used by RCMI Centers for inter-institutional collaborations and partnerships to further expand investigator access to scientific resources, to assemble mentoring teams to accelerate career development of junior investigators, and to foster community engagement for dissemination and implementation research.

### 3.1. How Have Inter-Institutional Resources Been Leveraged with National Research Programs?

At Tuskegee University (TU), RCMI funding catalyzed the NCI Comprehensive Partnerships to Advance Cancer Health Equity (CPACHE) grant, which resulted in a partnership with the University of Alabama at Birmingham CTSA. The National Center for Bioethics in Research and Health Care, at TU, has also been a major entry point for multi-institutional grants. This has led to making the local CTSA and other collaborating institutions more aware of the basic science expertise at TU. For example, in the initial CTSA grant, TU’s role was limited to bioethics. In the subsequent renewal, TU has had leadership roles in each of the CTSA cores.

The strategic approach used by Morehouse School of Medicine (MSM) to enhance collaborations with CTSA, Center for AIDS Research (CFAR) and other programs has been to brand itself as being an institution dedicated to advancing health equity. Additionally, MSM has leveraged similarities in programmatic structure (such as Administrative Core, Community Outreach Core, Research Core, and Training Core) to optimize administrative oversight and maximize economies of scale across inter-institutional collaborations. For example, MSM has used Centers for Disease Control and Prevention (CDC) funded Prevention Research Centers and other cores within other program grants to harmonize research efforts in the community, resulting in a proliferation of individual research projects, as well as leveraging infrastructure grants and partnerships. This approach has also fostered collaborations between basic scientists, clinical scientists and population scientists. Institutional resources provided via the shared research cores and the organizational administrative infrastructure of these program grants has facilitated junior investigator training, grant acquisition, community outreach, and faculty recruitment and retention. Additionally, MSM is beginning to expand its research focus to include rural communities, as dictated by an evolving overarching priority of the National Center for Advancing Translational Science (NCATS).

The CPACHE with the University of North Carolina (UNC) Chapel Hill was leveraged to establish the RCMI Center at North Carolina Central University (NCCU). After receiving the RCMI Center grant, the Duke CTSA committed $2 million of private funding (not contingent on its CTSA renewal) for NCCU to provide three elements in the partnership—Community Engagement, Pilot Funding, and Workforce Development. The change in interest was enhanced because NCATS leadership emphasized the involvement of partner institutions serving minority communities. 

Howard University and Georgetown University have collaborated in a CTSA, known as Georgetown-Howard Universities Center for Clinical and Translational Science (GHUCCTS). From the inception, the GHUCCTS has represented an equal partnership, with multiple principal investigators at Howard University and at Georgetown University. Most of the cores have leadership from both institutions and both Georgetown University and Howard University faculty members are among the Core directors. Howard University faculty members have access to Georgetown University resources, and vice versa. The leadership and organizational equality between Howard and Georgetown is illustrated by alternating steering committee meetings between the two institutions–done physically prior to Coronavirus Disease 2019 (COVID-19) and now done by a virtual communication platform.

### 3.2. How Do National Research Program Collaborations Help Promote Junior Investigators?

In general, the partnerships with national research programs have been beneficial for junior investigators’ career advancement. At NCCU, junior investigators are advised to be inquisitive and persistent in seeking mentors and collaborators. The leadership at TU takes a similar relentless approach in fostering the career development of their junior faculty. At the same time, the RCMI leadership and senior established investigators at TU serve as strong personal advocates to ensure that their young faculty members are treated with the same level of respect and are provided with the same quality of mentoring afforded to young faculty members from non-RCMI research-intensive majority institutions.

MSM has had similar experiences. Initially, the assumption was that investigators at the research-intensive, majority institution were properly mentoring the MSM junior faculty members. Instead, MSM junior investigators were often being marginalized and were not being properly mentored. In fact, they were being preferentially assigned to community-outreach activities, rather than developing their specific areas of research interest. Thus, the RCMI leadership and senior investigators must closely monitor the mentoring activities and assigned responsibilities to support their junior faculty.

Established in 2001, the mission of the NCI Center to Reduce Cancer Health Disparities (CRCHD) is to eliminate the unequal burden of cancer in the US through basic and community-engaged research and by diversifying the biomedical workforce for cancer research [9]. To establish the CRCHD at MSM, the Georgia CTSA was leveraged to show the depth of mentors. Additionally, by further leveraging the CRCHD and the RCMI Investigator Development Core, MSM was well-positioned to receive one NIH Mentored Career Development Award every other year from the Georgia CTSA and two candidates each year from the CRCHD. Thus, the CRCHD scholars doubled the size of the mentee pool, thereby enlarging the overall pipeline of trainees and contributing to the MSM RCMI Center’s growth.

The University of Puerto Rico Medical Sciences Campus (UPR-MSC) RCMI Center for Collaborative Research in Health Disparities (CCRHD) promotes participation of pilot-project awardees in the National Research Mentoring Network (NRMN), a nationwide consortium of biomedical professionals and institutions collaborating to provide all trainees across the biomedical, behavioral, clinical and social sciences, with evidence-based mentorship and professional-development programming. NRMN activities include guided virtual mentoring, coaching groups for grant-proposal writing, and research mentor and mentee training.

### 3.3. Have There Been Programmatic Challenges to Creating Synergy and Supporting Investigator Development in the National Networks?

Generally, inter-institutional collaborations and partnerships with CFAR, CTSA, Institutional Development Award (IDeA), Resource Center for Minority Aging Research (RCMAR) and other national research network programs have resulted in few programmatic, operational, fiscal, or reporting barriers. Administrative and fiscal arrangements have been straightforward and predictable. Collaborations have greatly expanded access to core facilities and more mentors and clinicians, resulting in enhanced grant success. While the advantages or benefits of inter-institutional collaborations with these programs have far outweighed the disadvantages, some RCMI grantees have encountered barriers when collaborating with individual research-intensive majority institutions (Table 1).

A significant barrier to the advancement of scientific research and investigator development is the limited participation of minority institution investigators in the mainstream collaborative networking venues organized by the NIH and other federal agencies. Increasingly, research is performed as team science within national research groups organized by disease entities (e.g., HIV, certain types of cancer) or by type of research (e.g., vaccine clinical trials) so that resources can be pooled and directed to enable studies of sufficient size to give meaningful data. Minority institutions have often been excluded from disease-specific networks because their applications cannot demonstrate adequate breadth and depth of scientific expertise and resources as found at larger research-intensive majority institutions.

Since research-intensive, majority institutions offer a critical mass of scientific investigators and more extensive resources and technologies, grant proposals from investigators at these institutions often appear more attractive during grant review. Having access to resources through national research networks may help address this issue. Participation in national research networks also provides access to discussions among the national thought leaders funded by NIH discipline-specific networks. Such discussions advantage participating scientists. The discussions help prioritize cutting-edge research for funding and setting public health policy. Mandating minority-serving institution inclusion in such national networks may facilitate broader institutional participation and better achievement of network inclusion and diversity goals.

### 3.4. What Were the Most Important Outcomes of Your Collaborations?

As measured by mentorship, conferences, publications, presentations, and/or other grant funding, outcomes from inter-institutional collaborations have been generally beneficial. For example, important outcomes of MSM collaborations as reported elsewhere have been new research awards, including training and collaborative center grants [10,11]. At UPR-MSC, an assessment of collaborations conducted by 55 RCMI-CCRHD investigators during FY2017–2019 revealed 168 unique collaborations, of which 25 were local, 112 national and 31 international. Investigators from other academic institutions were involved in 119 of the collaborations (71%). A total of 113 publications resulted from these collaborative efforts. More than half (51%) of the publications were from national collaborations, 36% from local collaborations, and 13% from international collaborations. During FY2017–2019, RCMI-CCRHD investigators collaborated in 79 studies supported by NIH grants. Many of these grants were related to health disparities affecting Puerto Rican and/or Hispanic populations or support research studies conducted by early stage investigators.

## 4. Discussion

In reviewing the breadth and scope of inter-institutional research collaborations, one is struck by how vital these collaborations are to increasing the visibility of the RCMI Centers and of the overall RCMI Program. NIMHD and other NIH Institutes and Centers frequently issue funding opportunity announcements that encourage participation by minority-serving institutions. However, RCMI grantee institutions often do not respond. A well-developed strategic plan to consistently submit joint grant applications incorporating multiple RCMI Centers would increase the much-needed visibility of RCMI Centers to NIMHD and other NIH Institutes and Centers. In this regard, it is imperative that the RCMI Centers not be pigeonholed or branded as being associated only with health disparities research. Developing a diversified research portfolio through inter-institutional collaborations with RCMI and non-RCMI research-intensive grantee institutions should be a key strategic goal.

Much can also be achieved by leveraging our resources as an RCMI community. While RCMI Centers commonly partner with research-intensive, majority institutions, how often do our RCMI Centers partner with each other to produce innovative research programs and responses to research funding notices? Minority-serving institutions bring a clear synergy to research-intensive, majority institutions, but larger inter-RCMI Center collaborations can also provide expanded and more inclusive populations. This is especially true when doing genomic research or any form of cohort research. For example, the COVID-19 pandemic has brought national awareness to the issue of health disparities which our communities face and represents an opportunity for RCMI Centers to collectively contribute.

Indeed, RCMI Centers can attract inter-institutional collaborations through their strong engagement with ethnic minorities and other underserved, vulnerable communities. Leveraging resources within (and across) the RCMI Community Engagement Cores should result in more multi-institutional research and grant applications. RCMI Centers also bring strength into the Investigator Development Cores, when investigative groups currently underrepresented in the sciences are an expected component of US federally funded research networks.

Additionally, RCMI Centers need to improve their marketing by emphasizing the strengths of their Research Infrastructure Cores. Many RCMI Centers have technical core facilities that are superior, both in terms of instrumentation and support personnel, to cores at some research-intensive, majority institutions. For example, Louisiana State University (LSU) investigators frequently use the core facilities at Xavier University of Louisiana (XULA). Thus, emphasizing the RCMI-supported technical cores is another means to increase visibility and desirability as sites for multi-institutional collaborative studies.

Aggressive exploration of potential partnerships with research-intensive, majority-serving institutions can be enhanced by meeting regularly with their research faculty and their institutional leadership. This requires persistence and perseverance, as well as close monitoring of NIH and other websites for grant announcements, especially those seeking minority-institution engagement. Ideally, partnership commitments with majority research institutions should be established shortly after the release of funding announcements or following the issue of notices of special interest. In this regard, creating registries of funded faculty at allied majority institutions will facilitate timely inter-institutional collaborative responses to grant announcements. RCMI Center leadership and RCMI investigators should know the research strengths and interests of their majority research-intensive institutional partners. Seizing opportunities to collaborate often depends upon RCMI Center leadership knowing where collaborations are possible and bringing investigators and concepts together quickly in response to funding announcements.

Although inter-institutional research collaborations often originate between established investigators, junior or early-stage investigators should be encouraged and guided to seek RCMI and non-RCMI investigator collaborators when forming their mentoring teams for K- and R-series NIH grant applications. Additionally, junior investigators should take every opportunity to participate in mentoring programs, such as Leading Emerging & Diverse Scientists to Success (LEADS) and NRMN. Moreover, whenever appropriate, promising junior investigators should be included in inter-institutional collaborations. This will also allow them to form life-long relationships with junior investigators at research-intensive, majority institutions and, at the same time, blur the lines between RCMI and non-RCMI grantee institutions. Similarly, when inviting senior investigators from partnering institutions or research networks to RCMI campuses for seminars, it is essential to allocate sufficient time for junior investigators to discuss their research interests and build mentoring opportunities with the guest speakers.

The importance of mentoring to address disparities in NIH grant awards going to minority investigators has been highlighted by Ginther and colleagues [12,13]. Recently, Hoppe and co-workers confirmed that “career stage and institutional resources influence the gap in the number of submissions by black and white researchers” [14]. They also noted that black applicants (as a group) were more likely to propose research topics that were less likely to be funded. Therefore, research topic choice is contributory. Only a small fraction of NIH funding directly supports health disparities or health population-type research. To advance minority investigators, RCMI Centers must not simply partner with majority institutions on health disparities projects. Instead, RCMI Centers need to selectively determine the type and focus of their investigators’ research and collaborations which will help accelerate the development of these investigators in their chosen scientific thematic areas. For example, encouraging junior investigators to do mechanistic studies complementary to health disparities will help the junior investigators become more competitive as independent investigators. Other suggested approaches to fostering collaborations and partnerships at RCMI Centers are provided in Table 2.

## 5. Conclusions

A workshop on inter-institutional collaborative research approaches, held in conjunction with the 2019 RCMI Program National Conference, explored strategies used by RCMI grantee institutions to strengthen and sustain collaborations with non-RCMI grantees. Such activities help to leverage federal resources, accelerate investigator development, and enrich community-engaged research. Similar to a recent report on CTSA institutional collaborations with community organizations, some factors worth addressing up-front when building inter-institutional collaborations include: partnership equity, communication, institutional policies and procedures, level of familiarity with varying fiscal and administrative processes, and financial management expectations [8].

## Figures and Tables

**Table 1:** Barriers to optimal synergy in collaborating with some research-intensive majority institutions.

• limited or no access to pilot project funding at the host majority institution
• uneven distribution of funds between minority and majority institutions
• lack of formal agreements to protect intellectual property in draft grant applications
• lack of clear guidelines to partnering agreements
• weak institutional commitment and support for collaborative efforts
• unspecified or absence of leadership roles for minority institution investigators
• limited availability of travel funds to pursue collaborations initiated online
• mismatched scientific resources and research emphasis between minority and majority institutions
• insufficient protected time for minority institution investigators to participate in collaborations and partnership planning sessions—including national committees
• infrequent face-to-face interactions because of significant geographic separation and time-zone differences

**Table 2:** Approaches to fostering collaborations and partnerships at RCMI Centers.

• provide investigators with protected time to participate in multi-disciplinary grant preparation working groups and study section participation
• monitor and disseminate funding opportunity announcements to investigators and research-intensive partnering institutions
• know strengths and weaknesses of partnering institutions and prospective collaborators
• invite outside experts as consultants and future collaborators
• leverage inter-RCMI Center shared expertise and resources to support synergistic participation in national research networks
• develop focused scientific expertise in areas beyond health disparities research
• emphasize ready access to underrepresented and other vulnerable communities
• market strengths of unique services offered by RCMI Center Research Infrastructure Cores
• use a virtual communication platform and other technologies to facilitate interactions between geographically dispersed institutions
• enlist visiting scholars and advisory committee members as mentors for junior investigators
• list updated scientific resources data in the eagle-i resource-discovery database
• list updated RCMI investigator data in Profiles research networking database
• monitor mentoring programs to ensure RCMI junior investigators get the support they need
• acknowledge participation in network collaborations as strengths in tenure and promotion applications
• provide travel assistance for investigators to meet with prospective mentors and collaborators or to learn highly specialized techniques

## Appendix A. Inter-Institutional Collaborations and Partnerships at Selected RCMI Centers

### Appendix A.1. Xavier University of Louisiana (XULA)

XULA has aggressively sought to form partnerships with local research-intensive institutions and health systems. To accomplish this, regular meetings have been scheduled with the Deans, Vice Presidents for Research, and senior officers of these institutions. The shared research focus on health disparities has facilitated faculty-to-faculty relationships, typically around potential funding opportunities. Additionally, XULA has engaged in statewide research development initiatives, such as those of the Louisiana Board of Regents.

One such collaboration has been the Louisiana Cancer Research Consortium (LCRC), which comprises members from XULA, Tulane University, LSU Health Sciences Center and Ochsner Medical Center. The goal of the consortium is to become a National Cancer Institute (NCI) designated cancer center. The state has invested approximately $13 million annually in the LCRC and constructed a $110-million LCRC Lab facility. These collaborations also led to developing a statewide trust fund with an endowment that now totals $600 million. Awards from this source include grants for enhancing research capacity and cost sharing on federal STEMM grants.

As a result of these partnerships, it has been possible to achieve substantial, long-term funding from such national programs as the CTSA of the National Center for Advancing Translational Science (NCATS), and the Institutional Development Award (IDeA) Networks of Biomedical Research Excellence (INBRE) of the National Institute of General Medical Sciences (NIGMS). These relationships also have led to the creation of state/city translational research/economic development organizations. With support from the city and state governments, “Biodistrict New Orleans” promotes research and economic development focused on the biosciences.

### Appendix A.2. University of Puerto Rico Medical Sciences Campus (UPR-MSC)

UPR-MSC comprises six schools: Medicine, Dental Medicine, Public Health, Pharmacy, Nursing, and Health Professions. Due to its geographic isolation from major continental US research centers, national collaborations and partnerships (examples below) are critically important for resourcing a robust research enterprise. The primary goal of the UPR-MSC RCMI Center for Collaborative Research in Health Disparities (RCMI-CCRHD) is to develop a vital infrastructure for conducting multidisciplinary collaborative research focused on health disparities in cancer; cardiovascular disease and metabolic syndromes, neurological disorders, and HIV/AIDS, which disproportionately affect Puerto Ricans, as well as other Hispanic populations. The RCMI-CCRHD also collaborates with other NIH-funded Programs at the UPR-MSC campus.

The Yale School of Medicine Transdisciplinary Collaborative Center for Health Disparities Research (YALE-TCC) collaboration focuses on precision medicine, especially in understanding the complex interplay between social factors and health disparities. As part of the collaboration, RCMI-CCRHD is a member of the YALE-TCC Consortium Core, which defines the overall governance and prioritization of research efforts through working groups. This collaboration also involves a partnership with CienciaPR, a nonprofit organization based at Yale University. It comprises scientists, professionals, students and citizens committed to advancing science in Puerto Rico and promoting science communication, science education, and scientific careers.

The University of Rochester CTSA collaboration focuses on community perceptions toward genomic research, which leverages a more than decade-long collaboration. Community perceptions toward genomic research are a priority area for future research, which requires rebuilding trust with the Puerto Rican community.

Administered by the University of Pittsburgh, Leading Emerging & Diverse Scientists to Success (LEADS) is an interactive online training program, which optimizes strategies for the professional development of post-doctoral fellows and junior faculty members from participating RCMI grantee institutions seeking to be competitive researchers.

### Appendix A.3. Clark Atlanta University (CAU)

In its 33rd year of funding, the RCMI program at CAU has played a significant role in developing biomedical research infrastructure and expanding inter-institutional collaborations. In 1999, the Center for Cancer Research and Therapeutic Development (CCRTD) was established with RCMI funding to conduct research on cancer biology and anti-cancer drug development. In 2004, the specific focus was directed to prostate cancer, which disproportionately affects African-American men. Over the past 16 years, the CAU faculty members have participated in many collaborative international prostate cancer research projects with scientists worldwide.

A significant partner is the Georgia Research Alliance (GRA), a private foundation created with funds provided by private industry and the State of Georgia, which was established in the early 1990s to increase the competitiveness of biomedical and technical research in the State of Georgia. GRA support has been critical for acquiring capital equipment and other infrastructure resources at the CCRTD over the last 15 years. Additionally, all GRA member universities have access to core facilities at other member institutions.

Last year, CCRTD signed a partnership agreement with the Georgia Cancer Center at Augusta University to promote collaborations in prostate cancer research, clinical trials and community outreach in Georgia. This partnership’s primary objective is to create a framework for cooperative and collaborative efforts between CAU and Augusta University thus providing an academic and clinical interchange between faculty members, students, staff, and administrators. This interchange will advance the collective understanding of prostate cancer’s etiology to ultimately serve and benefit the public good.

CCRTD is also in the process of creating a similar partnership with Cedars-Sinai Medical Center in Los Angeles, CA, to promote collaborations in the area of prostate cancer health disparities and student training. Other collaborations exist with the Veterans Administration Hospital in Los Angeles and the Fox Chase Cancer Center in Philadelphia, PA for the exchange of prostate cancer tissue and serum samples.

Finally, several CCRTD investigators are members of the African-Caribbean Cancer Consortium, promoting collaborations among scientists worldwide. Several CAU faculty members also have adjunct appointments at the Emory University-Winship Cancer Institute and the Integrative Cancer Institute at Georgia Tech. Due to these relationships, these faculty members share resources available in these Centers.

### Appendix A.4. North Carolina Central University (NCCU)

NCCU in Durham, North Carolina, was chartered in 1909 as the nation’s first public liberal arts institution founded for African Americans. Located in the Research Triangle, NCCU advances research in the biotechnological, biomedical, informational, computational, behavioral, social, and health sciences.

The RCMI Center at NCCU is a relatively new program (awarded in 2017). Largely because of the RCMI program, health disparities research has been recognized as a focus area for growth and investment at NCCU. As a commitment to the RCMI Center, NCCU has made a significant institutional investment of more than $500,000 over three years to establish the Health Equity, Environment and Population Health (HOPE) program in 2019. HOPE serves as a rural hub working in partnership with public health departments, community-based organizations, and other academic and private entities to reverse the adverse effects of social determinants of health and assure healthy communities through strategic partnerships reducing health disparities.

NCCU is part of the 17-institution UNC System and has been very active in developing partnerships with neighboring institutions. For example, NCCU has a U54 Comprehensive Partnerships to Advance Cancer Health Equity (CPACHE) grant in partnership with the Lineberger Cancer Center at UNC-Chapel Hill that leverages expertise and resources from both institutions to develop and enhance a robust cancer disparities program at both institutions. NCCU also has a similar CPACHE partnership with the Duke Cancer Institute at Duke University. North Carolina State University houses an NIEHS-funded Center for Human Health and Environment (CHHE). NCCU faculty members participate in the CHHE pilot grant program and use CHHE core facilities. NCCU has also established a partnership with RTI International receiving support for joint research projects and student internship opportunities. Such partnerships with neighboring institutions allow NCCU investigators access to cutting-edge core facilities, pilot programs, mentoring opportunities and subject-matter experts.

NCCU has also partnered with the Duke CTSA program, which has invested $2 million to foster joint research projects, training and certification of NCCU students in clinical research, and developing a robust community engagement program. This mutually beneficial program aims to produce a partnership—building trust in the community and developing careers in research—by leveraging both institutions’ strengths.

### Appendix A.5. University of Hawaii at Manoa (UHM)

UHM serves a student population comprising mainly Native Hawaiians, other Pacific Islanders, Filipinos, and multiple Asian ethnicities. Multiple academic units are engaged in biomedical, public health and socio-behavioral research. Located in the central Pacific Ocean, inter-institutional collaborations are crucial for complementing the small research faculty and programs based at UHM.

UHM is involved in several research networks. For example, it participates in the Mountain West Clinical and Translational Research–Infrastructure Network (CTR-IN) based at the University of Nevada, Las Vegas, one of 12 national NIGMS IDeA Networks for Clinical and Translational Research (IDeA-CTR). Additionally, the Hawaii IDeA Center for Pediatric and Adolescent Clinical Trials (HIPACT) participates as part of the IDeA States Pediatric Clinical Trial Network (ISPCTN), a network of IDeA sites established by the NIH’s Environmental Influences on Child Health Outcomes (ECHO) Program to recruit study participants from states whose populations are disproportionately rural and medically underserved. UHM is also part of the Northern Pacific Universities Global Health Research Training Consortium—providing a 12-month clinical research-training program for post-doctorate trainees and doctoral students in the health professions, sponsored by the NIH Fogarty International Center in partnership with other NIH Institutes and Centers.

The UHM Hawaii Center for AIDS (HICFA) has been a partner in the University of Washington/Fred Hutch CFAR for over 15 years. Inter-institutional collaborations also include the Native Alzheimer’s Disease Resource Center for Minority Aging Research (RCMAR) at Washington State University; the Johns Hopkins University School of Medicine’s Translational Research in Neuro-HIV and Mental Health (TRNAMH) course, which is funded by NIMH to provide mentoring and research opportunities to diversify mental health HIV research through innovative educational initiatives.

Substantial focus on health disparities is evident in almost all inter-institutional collaborative efforts within UHM. For example, the UHM NCI-designated cancer center encompasses several collaborations, including a US Affiliated Pacific Island cancer research and cancer control program with the UHM medical school and the University of Guam. More recently, the UHM RCMI Center, known as Ola HAWAII, was launched to grow a community of health disparities investigators and collaborators to address health disparities in the Pacific. Health disparities for indigenous Native Hawaiian and other Pacific Islander populations are apparent across multiple health conditions.

### Appendix A.6. Morehouse School of Medicine (MSM)

MSM is nationally recognized for its focus on increasing the quality and quantity of minority physicians and researchers. MSM is addressing culturally appropriate patient care and health disparities in Georgia’s underserved urban and rural populations. The National Center for Primary Care (NCPC) at MSM has the unique distinction of being the only congressionally sanctioned center in the US for community-oriented primary health care, with a particular focus on underserved populations and on the elimination of health disparities.

The Georgia Clinical and Translational Science Alliance (Georgia CTSA) is a statewide consortium of four institutions and affiliated health systems. The Georgia CTSA (formerly Atlanta CTSI) participates in the national CTSA consortium [15]. MSM leads the community engagement [16] and the Integrating Special Populations programs. The Georgia CTSA infrastructure extends the research capacity of RCMI core resources. Georgia CTSA has also expanded access to training and career development resources through NIH Scholars program slots and diversity supplements.

The MSM RCMI Center for Translational Research into Health Disparities (CTRHD) is building a sustainable framework for translational research by developing independently funded multi-disciplinary translational teams addressing health disparities in underserved communities. Collaborations and partnerships of the RCMI CTRHD include: (1) RCMAR program providing access to research development funds for MSM faculty members, as well as access to human and equipment resources; (2) Emory CFAR program providing pilot funding and a venue for faculty member recruitment; (3) Georgia Core Facilities Partnership (GCFP) networking biomedical technology and human expertise across Georgia’s academic institutions; and (3) the International Society on Extracellular Vesicles (ISEV) and member scientists who do microvesicle core research with CTRHD personnel.

The MSM CPACHE has enjoyed a long-standing regional partnership of over 15 years with the O’Neal Comprehensive Cancer Center at the University of Alabama at Birmingham (OCCCUAB) and TU, to address the burden of cancer among African Americans and other medically underserved populations in the southern US. The primary objectives of the partnership are to enhance institutional cancer research programs, to develop a pipeline of racial/ethnic minority investigators in cancer research at MSM and TU, and to increase cancer disparities research at OCCCUAB. As a direct result of this investment, MSM has developed a fully functional cancer program that includes a director and more than 30 research faculty members.

The NIH-funded Clinical Research Education and Career Development (CRECD) program provides funding for doctoral-prepared scientists at RCMI grantee institutions pursuing clinical and translational science careers. The program’s centerpiece is the Master of Science in Clinical Research (MSCR) at MSM and Master of Science (MSc) program at UPR-MSC. Both institutions have leveraged CRECD support to extend career development opportunities for junior investigators. A total of 97 MSc graduates from the UPR-MSC CRECD submitted 104 grants; 77 (74%) were funded at approximately $19.1 million, including three R01 awards [17].

The NIGMS funded National Research Mentoring Network (NRMN) aims to increase R-series grant success of underrepresented scientists [12,13]. The NRMN at MSM targets recruitment and training of investigators across the RCMI Consortium. The program has established an innovative Health Equity Learning Collaboratory to increase grant success of participants [18].
